# The relationship between health expenditure indicators and economic growth in OECD countries: A Driscoll-Kraay approach

**DOI:** 10.3389/fpubh.2022.1050550

**Published:** 2022-11-21

**Authors:** Umut Beylik, Umit Cirakli, Murat Cetin, Eyyup Ecevit, Osman Senol

**Affiliations:** ^1^Department of Health Management, Gulhane Health Sciences Faculty, Health Sciences University, Istanbul, Turkey; ^2^Department of Health Management, Faculty of Health Sciences, Izmir Bakircay University, Izmir, Turkey; ^3^Department of Economics, Faculty of Economics and Administrative Sciences, Tekirdag Namik Kemal University, Tekirdag, Turkey; ^4^Department of Economics, Faculty of Economics and Administrative Sciences, Erciyes University, Kayseri, Turkey; ^5^Department of Health Management, Faculty of Health Sciences, Karadeniz Technical University, Trabzon, Turkey

**Keywords:** health expenditures, economic growth, OECD countries, Driscoll-Kraay standard error approach, panel data analysis

## Abstract

**Introduction:**

The main purpose of the study is to examine the relationship between health expenditure indicators and economic growth in OECD countries.

**Methods:**

In this context, health expenditures and economic indicators data of 21 OECD countries were analyzed by the Driscoll-Kraay standard error approach within the scope of panel data analysis. While Gross Domestic Product (GDP) and income per capita were used as dependent variables, the amount of out-of-pocket health spending, per capita health expenditure, the amount of public health expenditure, the ratio of drug expenditures to gross domestic product, the share of current health expenditures in GDP were used as independent variables.

**Results:**

According to the results, in the model (Model 1) where real GDP level was used as the dependent variable, all health expenditure indicators were positively related to the economic growth. When the estimation results of Model 1 are examined, it is predicted that there will be an increase of 0.09% in GDP in case of a 1% increase in the share allocated to health services from GDP. In case of a 1% increase in the amount of out-of-pocket spending on healthcare, it is foreseen that there may be an increase of 0.04% in the real GDP. In the model (Model 2) where the per capita income variable is the dependent variable, it is seen that the increase in out- of-pocket health spending has a decreasing effect on the per capita income level, while the increase in public expenditures has an increasing effect on the per capita income level. From the findings of Model 2, it was found that if a 1% increase in the share of current health expenditures in GDP, there may be an increase of 0.06% in the amount of per capita income.

**Discussion:**

Concludingly, it is possible to say that that public resources allocated to health services play an important role in the economic growth.

## Introduction

Researchers and policy makers are particularly interested in achieving and sustaining economic growth and the factors affecting it. Solow (1) and Swan (2) focus on the neo-classical growth model of labor and capital, as well as the contribution of technological progress to economic growth. Following these authors, Nelson and Phelps (3) first mentioned the role of education in developing human capital to be able to apply new technologies. Romer (4) attaches importance to the development of human capital as a critical input in the generation of new ideas. While defining human capital, Shultz (5) sees health and education as the basic components of human capital and states that the development of human capital can be achieved with better education and health. Becker (6) points out that the main determinant of a country's development is how the country's individuals successfully utilize their talents, knowledge and health. In this context, the reason why health investments are important for the economic development of the country is related to the fact that these investments reduce the levels of disease and death and minimize human capital losses. According to the main argument of Bloom et al. (7); When health is considered as an important component of human capital, it also becomes a critical determinant of economic growth. Healthy individuals become physically and mentally more energetic and stronger. They can work more efficiently and earn more. They rarely leave their jobs due to illness.

Bloom et al. (8) demonstrate that a healthier and more productive workforce is important in the creation, adoption and application of new technologies, thereby supporting economic growth. The authors also point out that when health is neglected, investigating the relationship between human capital and economic growth cannot provide multifaceted results. Barro (9) investigates the effect of health capital on economic growth by adding health to the neo-classical growth model and draws a theoretical framework. Schultz (10) claims that health expenditures have a significant impact on productivity. Agenor (11) studies the optimal allocation of public health expenditures within the framework of an endogenous economic growth model. Here, it is explained in the theoretical framework that infrastructure and health have an impact on labor productivity and household utility. Due to these effects of health on the economy, especially on economic growth, it is also the subject of empirical studies in the health economics literature (12–17). However, since the findings are quite complex and not in harmony with each other, the investigation of this subject still continues as an important area of interest. In this study, the effect of health expenditure indicators on economic growth in OECD countries is analyzed. There are important reasons behind the selection of OECD countries. Namely:

When the empirical studies in the health expenditures-growth literature are examined, a type of health expenditure is generally taken as an independent variable and its effect on economic growth is analyzed. For example, Aboubacar and Xu (18) and Piabuo and Tieguhong (19) take per capita health expenditure as a measure of health expenditure, Wang (20) the share of health expenditure in GDP, and Zaidi and Saidi (21) total health expenditure as a measure of health expenditure. The most important difference of our study from the literature is that it analyzes the effects on economic growth in detail by including various types of health expenditures (such as per capita health expenditure, public health expenditure, out-of-pocket expenditure, share of health expenditures in GDP, and share of pharmaceutical expenditures in GDP) into the model. Thus, it will be able to present comprehensive empirical findings. Another contribution of the study to the literature is that it uses both per capita income and total GDP variables as a growth model, so that the effects on economic growth can be analyzed more soundly. The study uses second generation panel tests. The CADF test is used in unit root analysis, and the Driscoll-Kraay standard error approach is used in the estimation of long-term coefficients. Since the effects on economic growth are investigated by constructing two different regression models, it will be possible to obtain robust (soundly) results. In addition, comprehensive empirical findings on health expenditures will be able to make comprehensive recommendations for policies that will accelerate economic growth in OECD countries.

The main purpose of this research is to determine the effects of health expenditures on economic growth, both at the country level and at the individual level. In this direction, in accordance with the purpose of the research, individual and national health expenditures were included in the model as independent variables, while economic indicators at both individual and national levels were included in the models as dependent variables. This study may provide important contributions to literature. First, it includes both national and individual level indicators of health expenditures and economic growth, and this may provide better understanding of link between them. On the other hand, the method used in this study can make an important contribution to the literature related to the health expenditures and economic growth relationship. Because the Driscoll-Kraay estimator used in the study provides more robust results in the models with problems of cross-sectional dependence, autocorrelation and heteroscedasticity.

The following parts of the study can be stated as follows: The second part is a literature review. In the third part, the aim and scope of the empirical research, the model and the data set will be discussed. The fourth part includes the methodology used in the study and presents the findings. The study ends with a conclusion and policy recommendations.

## Literature review

Health is one of the important dimensions of human capital (22). A healthy population, in addition to being seen as the basis of national economic productivity and the assurance of economic growth, is an effective factor on labor supply and productivity by affecting the physical and mental conditions of employees. In the context of education and health, human capital is directly involved in the production function as a production input, as well as indirectly affecting other sources of economic growth such as technological development and physical capital accumulation (23). According to the report World Health Organization (24), the increase in health expenditures is a factor supporting the economic growth of both developing and developed countries. Poor health levels of individuals will cause a loss in workforce and productivity. This situation may negatively affect economic growth (25).

Another factor in explaining the relationship between health expenditures and economic growth is the effect of health on savings and investments. Good health can increase life expectancy and encourage individuals' motivation to save and invest in entrepreneurs. This may positively affect economic growth (8).

Health expenditures are seen as one of the top priority issues evaluated with different aspects in the health economics literature (13, 26–29). The literature primarily focuses on the determinants of health expenditures. Hitiris and Posnett (30) analyze the data of 560 countries and focus on the determinants of health expenditures. The findings indicate that the main determinant of health expenditures is economic growth. Thus, Ozturk and Topcu (31) identify a causality running from economic growth to health expenditures. Jack (32) shows that labor productivity is important to human capital investments, especially the physical and mental abilities of the workforce. Strauss and Thomas (33) prove the existence of a positive relationship between health and labor productivity. Toor and Butt (34) explore the issue for the Pakistani economy. The authors find that the main determinants of health expenditures are economic growth, urbanization, schooling rate, crude birth rate and foreign aid. On the other hand, Khan et al. (35) investigates the Malaysian economy with ARDL technique and concludes that health protection expenditures are determined by economic growth, population structure and population growth.

Behera and Dash (36) addresses the role of tax revenues and financial transfer as determinants of health expenditures. In this study, in which panel regression analyzes were performed, it is revealed that both variables have a positive effect on health expenditures. According to Abbas et al. (37) explores the socio-economic determinants of health quality for the Pakistani economy. The results of the research reveal that the quality of bureaucracy and accountability, health expenditures positively affect the quality of health, while the risk of population growth, socio-economic conditions, protectionism and out-of-pocket health payments decrease the quality of health.

Grossman (27) argues that a positive change in health investments will positively affect health outcomes in any society. Findings of Oladosu et al. (38) supports this view of Grossman. Oladosu et al. (38) analyze the impact of public health spending on health outcomes (such as infant mortality, malaria mortality, and maternal mortality) for Nigeria and Ghana. Contrary to the findings for Ghana, there is a positive relationship between public health expenditures and health outcomes in Nigeria.

Secondly, the literature explores the relationship between health expenditures and macroeconomic indicators (economic growth, productivity, etc.) (12, 14–16, 19, 21, 39–45).

Nobel laureate Fogel (12, 46), who investigated the effects of health on economic growth with a series of studies, found that three-thirds of economic growth could be explained by changes in health. Gyimah-Brempong (39), analyzing the relationship between health protection expenditures and economic growth in African countries, shows the existence of a positive correlation between the two variables. By analyzing the health-led growth hypothesis empirically, Bloom and Canning (13) show that the said hypothesis is valid and that there is a positive effect in the opposite direction, that is, from growth to health. Mayer (14) applies the Granger causality test to Latin American countries, indicating a causality from health expenditures to economic growth. Bloom et al. (7) investigates the effect of health on economic growth and shows the existence of a positive relationship between the two variables. Wang (42) examines the relationship between health protection expenditures and economic growth with panel regression analysis and panel quantile regression analysis for 31 countries. Research findings indicate that health protection expenditures increase economic growth in these economies. Chaabouni and Abdennadher (47) find a bidirectional causality between health expenditures and economic growth for the Tunisian economy with the help of Granger causality test.

Pradhan (41) focuses on the relationship between health spending and economic growth in 11 OECD countries. The results obtained from the panel data analysis indicate that there is a cointegration between the variables and a bidirectional causality. Eggoh et al. (15) examines the relationship between education, health and economic growth for African countries. According to the GMM estimation results, public education and health expenditures are in a negative relationship with economic growth. Using the panel GMM estimation technique, Chaabouni et al. (44) analyzes the relationship between health expenditures, CO_2_ emissions and economic growth for 51 countries. Empirical findings are that health expenditures are positively related to economic growth in the long run. This finding is similar to the findings of Narayan et al. (48) for 5 Asian countries and Hartwig (49) for 21 OECD countries. Applying the adaptive neuro-fuzzy technique, Mladenovic et al. (50) tries to estimate the impact of health protection expenditures on economic growth. The findings show evidence that the most influential factor in economic growth forecasts is health protection expenditures.

Çetin and Ecevit (51) investigates the link between health expenditures and economic growth for 15 OECD countries. Panel regression analysis reveals that there is no statistically significant relationship between health expenditures and economic growth. Piabuo and Tieguhong (19) focuses on African countries. Evidence from the OLS, DOLS and FMOLS estimates is that health expenditures are positively associated with economic growth in the long run. Applying ARDL model, Haseeb et al. (52) indicates that health expenditures are positively related with economic growth in the long run and there is a causal relationship from economic growth to health expenditures. Gok et al. (53) examines the impact of health expenditures on economic growth in emerging economies. Findings reveal a positive relationship between the variables. Erçelik (54) investigates the relationship between health expenditures and economic growth with the ARDL model in the Turkish economy. The study identifies a positive relationship between health expenditures and economic growth in the long run. Using panel ARDL and panel VECM causality tests, Zaidi and Saidi (21) explores the relationship between health expenditures, environmental degradation and economic growth for SSA countries. Panel causality analysis identifies a running causality running from health expenditures to economic growth.

Yang (16) analyzes the relationship between health expenditures, human capital and economic growth with the help of the panel threshold regression model for 21 developing country economies. Empirical findings, health expenditures affect economic growth negatively in developing countries with low level of human capital, health expenditures affect economic growth positively in developing countries with medium level of human capital, and health expenditures affect economic growth positively in developing countries with high level of human capital. Using ARDL bounds test and Toda-Yamamoto causality method for Australia, Kumar et al. (55) investigates the links among health expenditures, energy consumption and economic growth. Empirical findings show a u-shaped relationship between health expenditures and economic growth. Applying a panel GMM approach for selected African economies, Modibbo and Saidu (56) dwells on the relationship between health expenditures and economic growth. They find that health expenditures have a positive effect on economic growth. Raghupathi and Raghupathi (45) empirically examine the relationship between health protection expenditures and economic performance for the USA. Research findings indicate the existence of a positive correlation between health protection expenditures and economic growth and labor productivity.

Selvanathan et al. (57) analyzes the relationship between different government expenditures and economic growth for the Sri Lankan economy in the context of Wagner and Keynesian approaches. Findings from the ARDL approach indicate that health expenditures support economic growth in the long run. Yang et al. (58) examines the relationship between industrialization, economic growth, environmental degradation and health expenditures within the framework of the STIRPAT model. Panel causality test results provide evidence for bidirectional causality between health expenditures and economic growth. Ahmad et al. (17) analyzes the interrelationships between urbanization, health expenditures, environmental pollution and economic growth for the Chinese economy. In the study with the help of the system GMM approach, the existence of a mutually positive relationship between health expenditures and economic growth draws attention. Matahir et al. (59) intensifies on the links among energy efficiency, health expenditures and economic growth for Malaysia. VECM Granger causality analysis indicates that health expenditures and economic growth cause each other. Li et al. (60) explores the links between CO_2_ emissions, health expenditures and economic growth for BRICS countries. The study reveals that there is a causality running from economic growth to health expenditures for Brazil and South Africa. Applying a bootstrap ARDL technique for Saudi Arabia, Ageli (61) shows that there is a bi-directional causality between health expenditures and economic growth. Wu et al. (62) examines the link between health expenditures and economic growth in Asian countries by applying panel quantile technique. The results do not guarantee the existence of a positive relationship between the variables.

## Materials and methods

### Purpose of the research

The main purpose of this study is to examine the relationship between health expenditure indicators and economic growth in 21 OECD countries. In order to clearly reveal the relationship between the health expenditure indicators and the economic indicators, both individual and country level indicators were used in the analysis.

### Estimation strategy

The created panel data can be micro or macro according to the time they cover. Baltagi (63) stated in his study that panels up to 20 periods should be considered micro, and macro for panels with more than 20 periods. Since the time dimension of the variables considered within the scope of the research is 20 periods or more and falls into the macro panel class, the cross-sectional dependency states of the variables were first examined. The cross-sectional dependence states of the variables were tested with Breusch-Pagan CDLM1 and Pesaran CDLM2 and CDLM tests.

The stationarity of the series lies at the basis of the panel data analysis. Therefore, unit root tests are performed to examine the stationarity of the series. Second generation panel unit root tests should be used if data have cross-sectional dependence, whereby all units in the same cross-section are correlated. Cross-sectionally Augmented Dickey-Fuller (CADF) test of Pesaran (64) is one of the most preferred second generation panel unit root tests. The most preferred secondary generation unit root tests are Pesaran's cross-section augmented ADF (CADF) test and Im, Pesaran and Shin (CIPS) tests. In this study, CADF statistical values were calculated for unit root control in variables.

Findings obtained from models that do not provide basic assumptions are not free from errors. In this context, the first thing is to check whether there is a variable in the model that can cause multicollinearity problem. As stated by Gujarati (65), having multicollinearity problem in a model will cause incorrect predictor coefficients to be calculated. If there is such a problem in the model, corrective actions should be taken. Different tests and methods have been developed to detect this problem. One of these methods is the calculation of the Variance Inflation Factor (VIF) values of the variables. the VIF values of each variable are calculated using the formula (1/1-R2) (66). In the literature, it has been stated that acceptable VIF values can be accepted up to 4 in some studies, 5 and even 10 in some studies (67).

According to Joshi et al. (68), preliminary analyzes should be made about what the panel data model will be to be selected among the pooled model, fixed model and random model. F-test is conducted to select between the fixed-effect model and the pooled ordinary least square model in panel data analysis. Then, the Hausman test is used to determine the final model between fixed and random effect models (68).

Since the panel data have repetitive observations over time, there may be problems of cross-sectional dependence, heteroscedasticity and serial correlation. The inferences drawn from the panel data are not conclusive and the statistical result is completely biased if the presence of cross-sectional dependence in the model. Therefore, diagnostic controls about the problems of the cross-sectional dependence, heteroscedasticity and serial correlation in the model should made to check the model's validity (68). Tests applied for diagnostic controls in this study are Bhargava et al. Durbin-Watson and Baltagi-Wu LBI tests for autocorrelation, Modified Wald Test for heteroscedasticity, Breusch-Pagan LM Pesaran Scaled LM and Pesaran CD test for cross-sectional dependence.

In the case of autocorrelation, heteroscedasticity (changing variance), and cross-section dependence in a panel data analysis model, robust estimators should be used to overcome these problems. Joshi et al. (68) states that the White (1980) estimator, the Rogers (1993) estimator and the Driscoll and Kraay (69) estimator can be considered as the appropriate estimators to draw a conclusive result. However, as stated by Joshi, et al. (68) the Driscoll and Kraay estimator gives strong conclusive empirical results, and removes the deficits of the White and Rogers approach which produces inappropriate estimation when the cross-sectional dependence is present in the panel data set. Since it eliminates the effects of cross-sectional dependence problem, autocorrelation problem and heteroscedasticity problems in the developed models and enables us to reach more accurate estimator values, the Driscoll and Kraay estimator was used to estimate models.

### Model and data

Within the scope of the study, two dependent variables and five independent variables were used. The dependent variables are GDP and per capita income. The independent variables are the amount of out-of-pocket expenditure, per capita health expenditure, the amount of public health expenditure, the ratio of drug expenditures to gross domestic product, the share of current health expenditures in GDP. The type of data used is annual. The time dimension of the variables covers the periods 1990–2019. The data were obtained from the OECD database. In order to further generalize the results, all countries whose data can be accessed in line with the selected variables were tried to be included in the analysis. However, due to the availability of data, 21 countries were included in the study. The variables to be used in the model are shown in Table 1.

**Table 1:** Explanations of variables.

**Variables**	**Symbol**
Real gross domestic product	GDP
Per capita Income	PcINC
Per capita health expenditure	PcHEx
Public health expenditure	PHEx
Out-of-pocket health spending	OPHS
Share of current health expenditures in GDP	SCHEx
Ratio of drug expenditure in GDP	DEx

Descriptions and justifications of variables are given below:

Real Gross Domestic Product (GDP): It is an inflation-adjusted measure that reflects the value of all goods and services produced by an economy in a given year. Real GDP is expressed in base-year prices. GDP is the most used economic indicator when comparing incomes between countries. This indicator also has a positive effect on individual and social health status.

Per Capita Income: It is the most important economic indicator showing the level of development of a country. It is the value obtained by dividing the gross domestic product of the country by the population. Values are calculated in US dollars. As the per capita income level increases in a country, many social indicators, especially health indicators, show positive developments both at the individual and societal level. As Hu and Mendoza (70) stated, having a high per capita income level makes it easier to access health services. A higher income level allows individuals to spend more on their own health. A healthier individual can also participate in the workforce at an efficient level.

Health Expenditure Per Capita: It is one of the most important health indicators of a country. Health expenditure per capita is the amount of health expenditure per capita in US dollars. While economic developments at the individual and national level have an increasing effect on the amount of health expenditure per capita, individuals' spending on their own health has a positive effect on both labor productivity and general health level.

Public Health Expenditure: It is the amount of public expenditure in total health expenditures. The increase in public health expenditures facilitates people's free access to health services and enables them to meet their health needs more easily.

Out-of-Pocket Health Spending: It represents the direct payments made by individuals while receiving health services. However, the increase in the amount of out-of-pocket health expenditures creates an extra burden on households and increases inequality in the society.

Share of Current Health Expenditures in GDP: This ratio gives information about the amount of resources allocated to health services, according to other areas of use. By looking at the current health expenditure level of a country, it can be commented on its priority in the economy and the level of importance given to health.

Ratio of Drug Expenditure in GDP: This amount includes final expenditure on pharmaceuticals, wholesale and retail margins, and value added tax. Pharmaceutical expenditures, which have a significant share in total health expenditures, are in a mutual relationship with economic indicators. It is important to examine the relationship between pharmaceutical expenditures, which has a substantial proportion in total health expenditures, and economic indicators.

In the research, two different models will be obtained because each of the dependent variables will be produced separately. In the model, natural logarithmic transformation was applied in series with high numerical value. The equations of the models can be expressed as follows:

Model 1:


$$\begin{array}{l} {LNGDP}_{it}=\;C+\;\overset{pi}{\underset{j=1}{\sum }}\lambda_{ij}LNPcHEx_{i,t-j}+\;\overset{qi}{\underset{j=0}{\sum }}\delta_{ij}LNPHEx_{i,t-j}+\\ \;\overset{qi}{\underset{j=0}{\sum }}\varphi_{ij}LNOPHS_{i,t-j}+\;\overset{qi}{\underset{j=0}{\sum }}O_{ij}SCHEx_{i,t-j}+\overset{qi}{\underset{j=0}{\sum }}O_{ij}DEx_{i,t-j}+\\ \;\gamma_{1}LNPcHEx_{i,t-1}+\;\gamma_{2}LNPPHEx_{i,t-1}\;+\;\gamma_{3}LNOPHS_{i,t-1}\;+\\ \;\gamma_{4}LNSCHEx_{i,t-1}\;+\gamma_{5}DEx_{i,t-1}+\varepsilon_{it} \end{array}$$

Model 2:


$$\begin{array}{l} {LNPcINC}_{it}=\;C+\;\overset{pi}{\underset{j=1}{\sum }}\lambda_{ij}LNPcHEx_{i,t-j}+\;\overset{qi}{\underset{j=0}{\sum }}\delta_{ij}LNPHEx_{i,t-j}+\\ \;\overset{qi}{\underset{j=0}{\sum }}\varphi_{ij}LNOPHS_{i,t-j}+\;\overset{qi}{\underset{j=0}{\sum }}O_{ij}SCHEx_{i,t-j}+\overset{qi}{\underset{j=0}{\sum }}O_{ij}DEx_{i,t-j}+\\ \;\gamma_{1}LNPcHEx_{i,t-1}+\;\gamma_{2}LNPPHEx_{i,t-1}\;+\;\gamma_{3}LNOPHS_{i,t-1}\;+\\ \;\gamma_{4}LNSCHEx_{i,t-1}\;+\gamma_{5}DEx_{i,t-1}+\varepsilon_{it} \end{array}$$

The left sides of the equations represent the dependent variable. On the right side of the equations, “c” represents the constant variable, “α” represents the estimator coefficients of the independent variables, the “ε” represents the error term, “i” represents the cross-section, “Δ” represents differencing operator, “LN” represents logarithmic transformation and finally “t” represents the information about the period.

## Analysis results and discussions

The created panel data can be micro or macro according to the time they cover. Baltagi (53) stated in his study that panels up to 20 periods should be considered micro, and macro for panels with more than 20 periods. Since the time dimension of the variables considered within the scope of the research is 20 periods or more and falls into the macro panel class, the cross-sectional dependency states of the variables were first examined. The cross-sectional dependence states of the variables were tested with Breusch-Pagan CDLM1 and Pesaran CDLM2 and CDLM tests.

According to the results shown in Table 2, the H_0_ hypothesis that there is no cross-sectional dependence on variables has been rejected. In other words, there is cross-sectional dependence in variables. CADF test, which is one of the second generation unit root tests that take into account cross-sectional dependence, will be used test unit root in the series.

**Table 2:** Cross-sectional dependency results of variables.

**Variables**	**Breusch-Pagan**		**Pesaran scaled LM**		**Weigh CD**	
	**Statistics**	**Probability**	**Statistics**	**Probability**	**Statistics**	**Probability**
Real GDP	5458.01	0.000	255.05	0.000	73.76	0.000
Per capita income	2139.32	0.000	93.11	0.000	42.80	0.000
Per capita health Expenditure	2540.29	0.000	112.68	0.000	47.98	0.000
Public health expenditure	1665.86	0.000	70.01	0.000	35.83	0.000
Out-of-pocket health spending	290.80	0.000	2.91	0.003	5.71	0.010
Share of current health expenditures in GDP	3101.48	0.000	140.06	0.000	46.41	0.000
Ratio of drug expenditure in GDP	1082.85	0.000	18.63	0.000	13.94	0.000

In this study, CADF statistical values were calculated for unit root control in variables. According to the results of CADF test in the Table 3, it is understood that the series are stationary. After the stationary conditions of the variables are determined, the Variance Inflation Factor (VIF) values of the variables will be calculated to se whether there is multicollinearity problem in data.

**Table 3:** CADF panel unit root test.

**Variables**	**t-bar**	**Cv10**	**Cv5**	**Cv1**	**Z(t-bar)**	**P**
Real GDP	−1.342	−2.070	−2.150	−2.300	1.907	0.972
Per capita income	−1.905	−2.040	−2.110	−2.230	−2.971	0.001
Per capita health expenditure	−2.785	−2.060	−2.150	−2.350	−7.268	0.000
Public health expenditure	−2.966	−2.070	−2.150	−2.300	−5.355	0.000
Out-of-pocket health spending	−3.216	−2.070	−2.150	−2.300	−6.8550	0.000
Share of current health expenditures in GDP	−2.258	−2.070	−2.150	−2.300	−2.374	0.009
Ratio of drug expenditure in GDP	−1.959	−2.070	−2.150	−2.300	−0.979	0.169

t-bar test, N, T = (21, 26); Obs = 523, Augmented by 2 lags (average).

As seen in Table 4, each variable in the model was made a dependent variable once and the value of R^2^ was obtained. The VIF values of the variables were calculated using the specified formula and the values in the table were obtained. The most critical value of the variables is the coefficient of 5. Since the VIF value of the variables used in the research <5, there is no variable in the model that can cause multicollinearity problem. The next step in the panel data is to determine which approach the model will be determined by. Within the three basic panel data analysis approaches, it is necessary to determine which model to be developed is the most appropriate. Results of the panel data model determination tests are shown in Table 5.

**Table 4:** VIF values for variables.

**Variables**	***R* ^2^**	**VIF value**
Real GDP	0.43	1.75
Per capita income	0.60	2.50
Per capita health expenditure	0.34	1.51
Public health expenditure	0.29	1.40
Out-of-pocket health spending	0.47	1.88
Share of current health expenditures in GDP	0.25	1.33
Ratio of drug expenditure in GDP	0.17	1.20

**Table 5:** Panel data model identification tests.

	**Model 1(GDP)**		**Model 2 (Per capita Income)**	
	**Statistic**	**p**	**Statistic**	**p**
F- Fixed Effects	43.89	0.000	27.34	0.000
Hausman Test	17.64	0.003	6.83	0.23

First, the validity of the pooled model was tested by F test, the H_0_ hypothesis was rejected, and the fixed effects approach was found to be valid. In the next step, it is necessary to determine whether the fixed effects approach or the random effects approach is valid in the model. In order to make this determination, the Hausman test was performed. The results of the Hausman tests shows that the most appropriate approach for Model 1 (GDP) was the fixed effects approach. On the other hand, the random effects approach is valid for Model 2 (PcINC). After determining the approach with which the models will be predicted, autocorrelation, heteroscedasticity and cross-sectional dependency tests should be checked in the models to see whether there are problems in the basic assumptions of panel data. If there are problems, then corrective robust estimators will be used to solve them.

To reach the most accurate results in the model developed in the panel data modeling studies, the first factor to be considered is to check whether there is an autocorrelation problem in the model. According to the results of the autocorrelation test in Table 6, it was found that there is an autocorrelation problem in the Model 1 since the test values are less than 2. On the other hand, In Model 2, the autocorrelation coefficients > 2 indicate no autocorrelation problem in the Model 2.

**Table 6:** Results of autocorrelation, heteroscedasticity, and cross-sectional dependence tests.

	**Tests**	**Model 1 (GDP)**		**Model 2 (per capita income)**	
**Autocorrelation tests**	Bhargava et al. Durbin-Watson	0.32		2.27	
	Baltagi-Wu LBI	0.46		2.31	
**Heteroscedasticity test**	Modified Wald Test	**Chi** ^**2**^	***p***	**Chi** ^**2**^	***p***
		3462.14	0.000	2506.18	0.000
**Cross-sectional dependence tests**		**Statistic**	**p**	**Statistic**	**p**
	Breusch-Pagan LM	3145.17	0.000	2493.18	0.000
	Pesaran Scaled LM	256.39	0.000	157.82	0.000
	Pesaran CD	31.89	0.000	19.73	0.000

Another aspect that needs to be tested in the model is the control of the changing variance state (heteroscedasticity). Modified Wald test used to test heteroscedasticity problem. From the results of Modified Wald test, it is seen that there is a problem of variance changing in the models. Therefore, this problem needs to be corrected by using robust estimators.

Another assumption that should be considered in the panel data models is to check whether there is a cross-sectional dependency problem in the models. Cross-sectional dependency states of the models were checked with three different test types. In all three test types, it is seen that the models have the cross-sectional dependency problem and need to be corrected by using robust estimators. In this context, Driscoll-Kraay standard error approach, which provides solution to previously mentioned problems, was used to estimate panel data.

When the situation of meeting the basic assumptions of the developed models is examined, it is seen that there are problems of correlation, changing variance (heteroscedasticity) and cross-sectional dependence in the Model 1. Panel data model identification tests were revealed that the most appropriate approach for the Model 1 was the fixed effects approach, while the most appropriate approach for the Model 1 was the random effects approach for the Model 2. For Model 2, it is seen that although there is not autocorrelation problem, there are changing variance and cross-sectional dependency problem in the model. Therefore, the Driscoll Kraay Standard Error estimator was used to eliminate errors that may occur due to these problems. Estimation results for the models are shown in Table 7.

**Table 7:** Estimation results of Driscoll-Kraay standard error.

**Period:1990–2019, cross section: 21, total number of observations: 541**					
	**Variable**	**Coefficient**	**Drisc/Kraay**	***t***	***p***
**Model 1 (GDP)**	Share of current health expenditures in GDP	0.097	0.017	4.47	0.000
*R*^2^: 0.27	Ratio of drug expenditure in GDP	0.868	0.236	1.01	0.322
F-statistic: 15.37	Out-of-pocket health spending	0.041	0.012	3.46	0.002
Prob (F-Statistic): 0.000	Public health expenditure	0.078	0.007	3.79	0.001
	Per capita health expenditure	0.047	0.009	5.00	0.000
	C	24.220	0.385	62.87	0.000
**Model 2 (Per capita Income)**	Share of current health Expenditures in GDP	0.066	0.001	14.05	0.000
*R*^2^: 0.34	Ratio of drug expenditure in GDP	−0.258	0.006	−1.23	0.218
F-statistic: 6.32	Out-of-pocket health spending	−0.270	0.001	−7.26	0.000
Prob (F-Statistic): 0.000	Public health expenditure	0.036	0.0003	9.24	0.000
	Per capita health expenditure	−0.023	0.001	−23.41	0.000
	C	2.507	1.539	6.82	0.000

According to the estimation results of Driscoll-Kraay Standard Error shown in Table 7, it is seen that the Model 1 is significant at the level of 1% significance. When *R*^2^ is examined, it can be said that In the Model 1, the percentage of independent variables explaining the dependent variable is 27%, and this level of explanation is sufficient.

When the estimation results of Model 1 are examined, it is seen that the share of current health expenditures in GDP is positively correlated with GDP. In other words, the share of current health expenditures in GDP increases economic growth. This can be interpreted that there will be an increase of 0.097% in economic growth in case of a 1% increase in the share allocated to health services from GDP. The results indicate that out-of-pocket health spending is positively affects GDP. This means that that out-of-pocket health spending encourages economic growth. In case of a 1% increase in the amount of out-of-pocket spending on healthcare, it is foreseen that there may be an increase of 0.041% in economic growth. The results also indicate that public health expenditure is positively related to GDP. This implies that public health expenditure stimulates economic growth. From the results, it is detected that if there is an increase of 1% in the amount of public health expenditure, it is foreseen that there may be an increase of 0.078% in economic growth. The coefficient of health expenditure per capita is positive and statistically significant at 1% level. This means that health expenditure per capita raises economic growth. If there is a 1% increase in the amount of health expenditure per capita, it is predicted that there may be an increase of 0.047% in economic growth. Finally, it is found that the coefficient of drug expenditure is positive but no statistically significant. This implies that there is no statistically significant relationship between the amount of drug expenditure and economic growth. As can be seen from the results of estimation for the Model 1, all independent variables are positively related to GDP level. In other words, the realization of increases in independent variables (health expenditure indicators) supports the country's growth by contributing positively to the country's economy.

When the findings related to the Model 2 are examined, it is found that the rate of GDP allocated to health services is positively correlated with GDP. This implies that the rate of GDP allocated to health services raises economic growth. If a %1 increase in the rate of GDP allocated to health services occurs, there may be an increase of 0.066% in economic growth. In this model, the coefficient of the ratio of drug expenditures in GDP is negative but no statistically significant. This means that there is no statistically significant relationship between the ratio of drug expenditures in GDP and economic growth. The findings reveal that out-of-pocket health expenditure is negatively related to GDP. This implies that out-of-pocket health expenditure decreases economic growth. In other words, it is foreseen that if there is an increase of 1% in the amount of out-of-pocket health expenditure, there may be a decrease of 0.270% in economic growth. The findings also reveal that public health expenditure is positively affects GDP. This means that public health expenditure increases economic growth. In other words, it is estimated that an increase of 1% in the amount of public health expenditure may result in an increase of 0.036 % in economic growth. It is detected that the coefficient of health expenditure per capita is negative and statistically significant at 1% level. This implies that health expenditure per capita reduces economic growth. In the event of an increase of 1% in the amount of health expenditure per capita, it is foreseen that there may be a decrease of 0.023% in economic growth.

Our finding is that there is a positive relationship between public health expenditure and economic growth coincides with the result of Eggoh et al. (15). This study reveals that public health expenditure encourages economic growth. Our finding does not coincide with the results of Yang (16). This study shows both positive and negative relationship between the variables. Our finding proving the positive relationship between health expenditure per capita and economic growth is similar to the findings of Chaabouni et al. (43) and Aboubacar and Xu (18). The authors find that health expenditure per capita is positively related to economic growth. Our finding on the positive relationship between the share of health expenditure in GDP and economic growth is compatible with the results of Wang (20) and Narayan et al. (48). These papers indicate a positive link between the variables.

## Conclusion and recommendations

The main purpose of this study was to examine the relationship between health expenditure indicators and economic growth in 21 OECD countries. In this context, annual data of health expenditure indicators and economic indicators between 1990 and 2019 of 21 OECD countries were analyzed using Driscoll-Kraay Standard Error approach within scope of panel data analysis. In the study, variables of the GDP and per capita income were used as the dependent variables, while the variables of the amount of out-of-pocket expenditure, per capita health expenditure, the amount of public health expenditure, the ratio of drug expenditures to gross domestic product, the share of current health expenditures in GDP were used as the independent variables. Two different models have been developed for two dependent variables.

From the estimation results for Model 1, it was found that the variables of the share of current health expenditures in GDP, out-of-pocket spending on healthcare, public health expenditure, health expenditure per capita were significantly positively related to GDP. On the other hand, it was found that there was no significant relationship between drug expenditure and GDP. Although there are different factors that increase economic growth, these results for Model 1 (GDP) show that increase in the health expenditures both at the individual level and at the national level will contribute to the economic growth at national level. In other words, it is seen that expenditures on health services have a positive increasing effect on economic growth.

In the Model 2 where the per capita income is the dependent variable, it is seen that the increase in individual level expenditures has a decreasing effect on the per capita income level, while the increase in public expenditures has an increasing effect on the per capita income level. This result can be interpreted as that public health expenditures have a significant supportive effect on economic growth. When examined in general, it is seen that each resource allocated to health services is seen as an investment and that these expenditures indirectly support economic growth. It is thought that the comparison of the results by examining these indicators on other country groups will contribute to the literature.

Individual and national public health expenditures also have an impact on economic growth, especially on the general health level of the society. As the amount of spending on health services in a society increases, infant mortality decreases, the average life expectancy increases, and the society can be healthier and more productive. On the other hand, investing in the public expenditures for health programs also function as macroeconomic stabilizers. Of course, although the increase in health expenditures does not always mean better health, it can be expected that the increase in the health expenditures, especially governmental health expenditures, may contribute to improving the health of vulnerable people. As a result, healthy people will contribute more to the growth of the economy by being more productive. In this context, this study shows that that investments in health services support economic growth.

This study has some limitations. First, only countries whose data are available within OECD countries are included in the sample. Therefore, it is not possible to generalize the results to all OECD countries. Secondly, due to the insufficient level of data on the variables, the time dimension of the research had to be limited. Third, apart from health expenditures, there are many factors affecting economic growth. Thus, the results are valid under the assumption of other factors ceteris paribus. As a suggestion within the scope of the research, new models can be produced by adding different indicators to models. Results can be compared across different country groups under similar indicators. In order to reveal how and at what level health expenditures affect the income levels of the countries, research can be done with dynamic panel data threshold models.

## Data availability statement

Publicly available datasets were analyzed in this study. This data can be found at: https://stats.oecd.org/.

## Author contributions

UC, EE, and MC organized and provided feedback on the writing of the manuscript. All authors contributed to the conception and design of the study, wrote the first draft of the manuscript, wrote sections of the manuscript, contributed to manuscript revision, read, and approved the submitted version.

## Conflict of interest

The authors declare that the research was conducted in the absence of any commercial or financial relationships that could be construed as a potential conflict of interest.

## Publisher's note

All claims expressed in this article are solely those of the authors and do not necessarily represent those of their affiliated organizations, or those of the publisher, the editors and the reviewers. Any product that may be evaluated in this article, or claim that may be made by its manufacturer, is not guaranteed or endorsed by the publisher.
